# Deep-Learning Based Label-Free Classification of Activated and Inactivated Neutrophils for Rapid Immune State Monitoring

**DOI:** 10.3390/s21020512

**Published:** 2021-01-13

**Authors:** Xiwei Huang, Hyungkook Jeon, Jixuan Liu, Jiangfan Yao, Maoyu Wei, Wentao Han, Jin Chen, Lingling Sun, Jongyoon Han

**Affiliations:** 1 Key Laboratory of RF Circuits and Systems, Ministry of Education, Hangzhou Dianzi University, Hangzhou 310018, China; hduljx@hdu.edu.cn (J.L.); yaojiangfan@hdu.edu.cn (J.Y.); lucienwell@hdu.edu.cn (M.W.); wentaohan@hdu.edu.cn (W.H.); chenjin272@hdu.edu.cn (J.C.); sunll@hdu.edu.cn (L.S.); 2 Research Laboratory of Electronics, Massachusetts Institute of Technology, Cambridge, MA 02139, USA; hkjeon@mit.edu (H.J.); jyhan@mit.edu (J.H.); 3 Department of Electrical Engineering and Computer Science, Massachusetts Institute of Technology, Cambridge, MA 02139, USA; 4 Department of Biological Engineering, Massachusetts Institute of Technology, Cambridge, MA 02139, USA

**Keywords:** label-free, white blood cell classification, deep learning, transfer learning, neutrophil activation, point-of-care

## Abstract

The differential count of white blood cells (WBCs) is one widely used approach to assess the status of a patient’s immune system. Currently, the main methods of differential WBC counting are manual counting and automatic instrument analysis with labeling preprocessing. But these two methods are complicated to operate and may interfere with the physiological states of cells. Therefore, we propose a deep learning-based method to perform label-free classification of three types of WBCs based on their morphologies to judge the activated or inactivated neutrophils. Over 90% accuracy was finally achieved by a pre-trained fine-tuning Resnet-50 network. This deep learning-based method for label-free WBC classification can tackle the problem of complex instrumental operation and interference of fluorescent labeling to the physiological states of the cells, which is promising for future point-of-care applications.

## 1. Introduction

White blood cells (WBCs) are important immune cells in the human body, which are mainly involved in immune response and regulation of inflammation [1]. The three-type differential count of WBCs, i.e., monocyte, lymphocyte, and granulocyte, provides an important indication for the diagnosis of many diseases, such as leukemia [2,3] and cancers [4]. As the dominant fraction of granulocytes, neutrophils usually show an abnormal change in the expected number when dealing with damaged tissues and unresolved infections [5]. The transition from an inactivated state to an activated state is a basic condition for neutrophils to function as immune cells. Therefore, the rapid classification and counting for activated and inactivated neutrophils based on the three-type WBC classification can provide an important reference for human immune state monitoring.

Traditionally, the manual method of WBC classification and counting is carried out in clinics or laboratories by well-trained professionals. They usually stain the WBCs’ nuclei and then perform detection, classification, and counting according to the morphological difference of the nucleus [6]. However, there are some disadvantages to the manual method. First, the required manual operation by professionals [7] and the staining process is cumbersome. Manual classification of WBCs can cause errors, including sampling errors or accuracy errors due to statistical probabilities. Second, the staining process may change the physiological states of WBCs, which would cause changes in their morphologies. WBCs’ physiological states may be transformed from inactivated to activated so that their morphological features will be different from the original ones. On the other hand, automated analysis equipment, such as the Coulter Counter and Flow Cytometer, is also commonly used for classification, which is based on electrical characteristics or laser scattering characteristics, respectively. The disadvantages of automated analysis equipment are the high cost of the instrument and low portability [8,9,10].

To tackle the deficiency of manual methods and automated analysis equipment, intelligent image processing methods are applied to WBC classifications. The basic principle of image processing is to obtain the characteristic information from the WBCs images and then classify them automatically. Image processing generally can be divided into the following steps: datasets generation, pretreatment, feature extraction, and classification [11]. In particular, the recent advance of deep learning techniques has provided many powerful image processing solutions. Since the advent of Alexnet in 2012 that refreshed the record of image classification [12], deep learning based blood cell image analysis has become a hot research topic [13].

For example, Qin et al. proposed one fine-grained WBC classification framework that can classify the stained WBCs into 40 sub-categories using a self-built deep learning network. The WBC sample needs to remove the red blood cells (RBCs) in the background and then segmented each WBC into a single frame [14]. In another work, M. S. Wibawa [15] applied a simple 5-layer deep learning Convolutional Neural Network (CNN) to classify stained WBC images into four categories and compared them with machine learning methods, namely Support Vector Machine (SVM), K-Nearest Neighbor (KNN), and Multi-Layer Perceptron (MLP) classifiers, to classify the feature values of WBCs, such as average brightness, entropy, kurtosis, etc. Zhao et al. extracted feature values through texture features then used the SVM classifier to divide stained WBCs into Basophil, Eosinophil, and others [16]. They finally used deep learning to extract the feature values of others and used the random forest method to classify them. However, WBC datasets in the above studies are all three-channel RGB images obtained after fluorescent staining. The staining materials affect the physiological state and morphology of WBCs, which would lead to inaccurate WBC classification. Furthermore, the WBC staining process impedes the rapid point-of-care (POC) detection application. To avoid the adverse effects of staining reagents on cell viability and cell signal transduction, label-free WBC classification is of great significance.

So far, some label-free WBC classification work has been proposed in the literature and verified the feasibility of the label-free classification method. For example, Y. Li et al. proposed a lensless holographic imaging method to capture label-free WBC images and classify them based on machine learning [17]. They selected morphological features, such as cell ridge, cell diameter, etc., for holographic WBC images based on the analysis of variance (ANOVA). The over-fitting problem was reduced to a certain extent, and the feature values were classified and compared through different machine learning methods (SVM, random forest, etc.). Moreover, T.-F. Wua et al. used a microfluidic system to obtain the forward scattering of cells, and the different feature values of forwarding scattering were classified by SVM to achieve label-free classification [18]. The microfluidic system hardware facilities used in the article can obtain cell volume, membrane capacity, and optical properties of cells by measuring electrical signals, optical signals, etc., and the label-free automatic classification of cells can be performed based on these features. However, there are still challenges remaining for the existing label-free methods. First, since machine learning requires specific screening of the WBC biophysical features and manually selecting appropriate features as learning data through thresholds, the accuracy of feature selection may be limited for existing methods. Second, the existing label-free WBC classification system generally needs custom-designed hardware systems [17,18]. Using the commercially unavailable hardware to automatically classify cells is troublesome in the manufacturing process and may produce deviations due to the inaccurate manufacturing of microfluidic components.

Therefore, this paper proposed a label-free WBC classification method based on deep learning to tackle the above-mentioned challenges. The novelty and contributions of our method compared with the existing label-free methods are as follows: Our label-free WBC images are directly captured by an off-the-shelf microscope when WBCs flow through a simple microfluidic channel and are used as training datasets for classification. No manual feature selection or complicated microfluidic device design is required. Considering that the feature extraction of label-free WBC images is different from fluorescently labeled WBCs, Resent-50 with short connection structures was employed and trained to reach a high accuracy of around 90%. Based on the deep transfer learning model, the classification and counting of activated and inactivated neutrophils were further realized based on morphological features of aspect ratio and roundness. The proposed method enables highly convenient and accurate WBC identification and minimizes the disturbance of the staining process to the WBCs. Hence, it is promising for future label-free and rapid immune state monitoring.

## 2. Materials and Methods

### 2.1. WBC Datasets Generation

**Figure 1:**
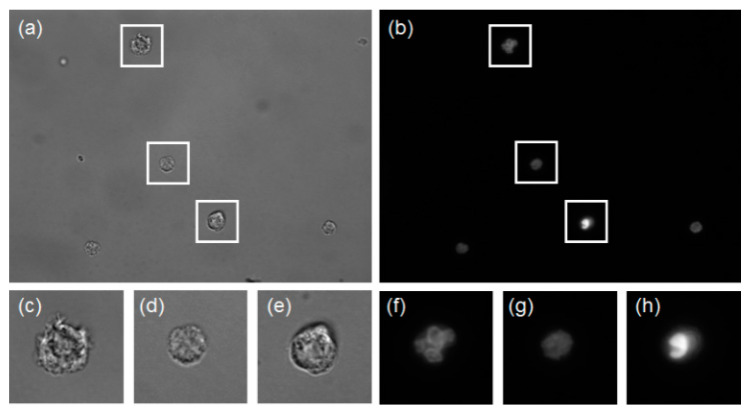
White blood cells (WBC) images taken by microscope (**a**) bright-field WBC images, (**b**) the corresponding fluorescent WBC images, (**c**–**e**) bright-field granulocyte, lymphocyte, and monocyte images, (**f**–**h**) the corresponding fluorescent granulocyte, lymphocyte, and monocyte images.

As the proportion of RBCs in the whole blood is too large compared with WBCs, the WBCs in whole blood samples cannot be directly distinguished through microscope observation. Separating WBCs from RBCs is an essential step in generating WBC datasets. This paper uses a label-free method based on the spiral microfluidic channel to separate WBCs. Compared with general density gradient separation, spiral inertial separation has the advantages of high throughput, fast processing, low cost, and portability, and it can also reduce the activation rate of WBC in vitro to avoid the impact on the test results [19]. A more detailed spiral microfluidic channel discussion is beyond the scope of this article. With spiral microfluidics, the WBCs were separated from peripheral blood samples that were collected from healthy donors who reported general health and no use of medical prescriptions in the last 2 weeks before enrollment and were purchased from Research Blood Components, LLC (Boston, MA, USA). The separated WBC were fluorescently stained, then bright-field and fluorescent imaging were sequentially performed in the same field-of-view area through a microscope with 100× magnification objective lens. Fluorescent imaging can show the details of WBC nucleolus features, and they are used to distinguish the actual type of each corresponding bright-field WBC image. Then the label-free bright-field WBC images with known type were used to form the training or testing datasets for Resnet-50 model. The original image and the fluorescence label are shown in Figure 1a,b.

### 2.2. Datasets Preprocessing

**Figure 2:**
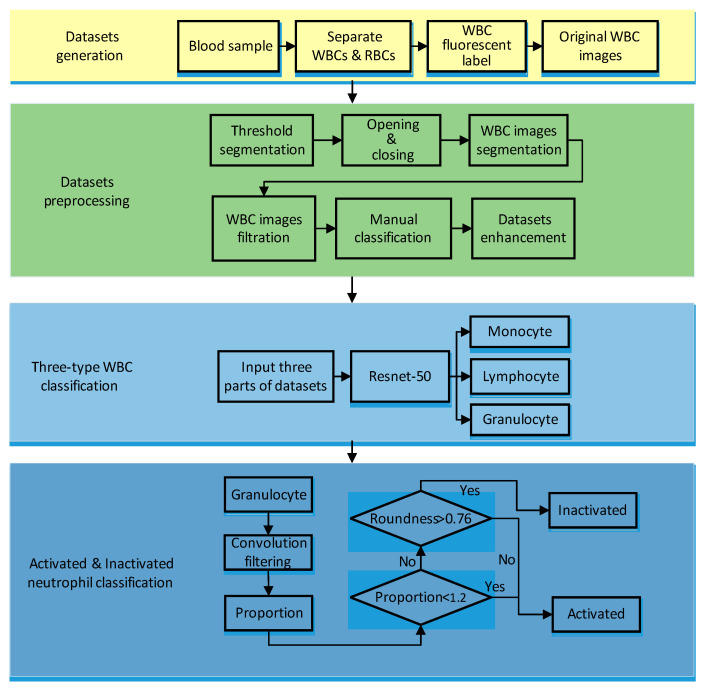
Whole processing flowchart for WBC classification.

The preprocessing flow is shown in Figure 2. This paper used the threshold segmentation method to achieve WBC and background segmentation. Threshold segmentation methods are widely used in image analysis applications where a significant gap between the target and background pixel gray levels exist.

We employed the Otsu [20] method of adaptive threshold and obtained the threshold value in (1),

(1) $$\;g=\;\omega_{1}*\;\omega_{2}*{\left(\mu_{1}-\mu_{2}\right)}^{2}$$

where g represents the final threshold value, $\omega_{1}$ represents the proportion of background pixels, $\omega_{2}$ represents the proportion of foreground pixels, $\;\mu_{1}$ represents the average grayscale value of the background, $\mu_{2}$ represents the average grayscale value of the foreground.

**Figure 3:**
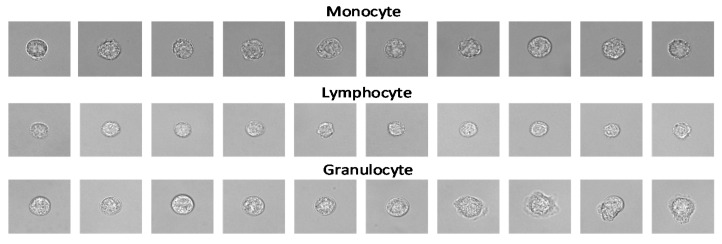
Examples of three-type WBC images in the datasets after segmentation.

After threshold segmentation, the open operation and close operation were performed on the WBC images to make the cell contour clear. Then the cell images were segmented after determining the specific coordinates of the cell according to the cell contour. We screened the segmented WBC results to eliminate the cells on the edges or multiple adhesions to ensure that there was only one WBC in the center of an image, which is more favorable for the extraction of WBC feature values. After that, WBCs were classified manually according to their corresponding fluorescent labels, and finally, three types of WBC datasets (granulocyte, lymphocyte, monocyte) were achieved. Among them, granulocytes include basophil, neutrophil, and eosinophil. The datasets after segmentation are shown in Figure 3.

From Table 1, it can be seen that the number of various WBCs was unbalanced because the number of WBCs in the body is unbalanced. To improve the accuracy and reduce over-fitting, enhanced processing of different types of WBC datasets is necessary. This paper used rotation and flipping methods (other methods will affect the details of WBCs) to amplify various types of cells to about 10,000. This method will produce a black frame. To reduce the impact of the black frame on the accuracy, we used the interpolation method to enlarge the cell picture. After the rotation, we uniformly intercepted the 200 * 200 picture without a black frame. Finally, the numbers of WBCs used after the balancing process was obtained, as shown in Table 1.

### 2.3. Deep Neural Network Classifier

**Figure 4:**
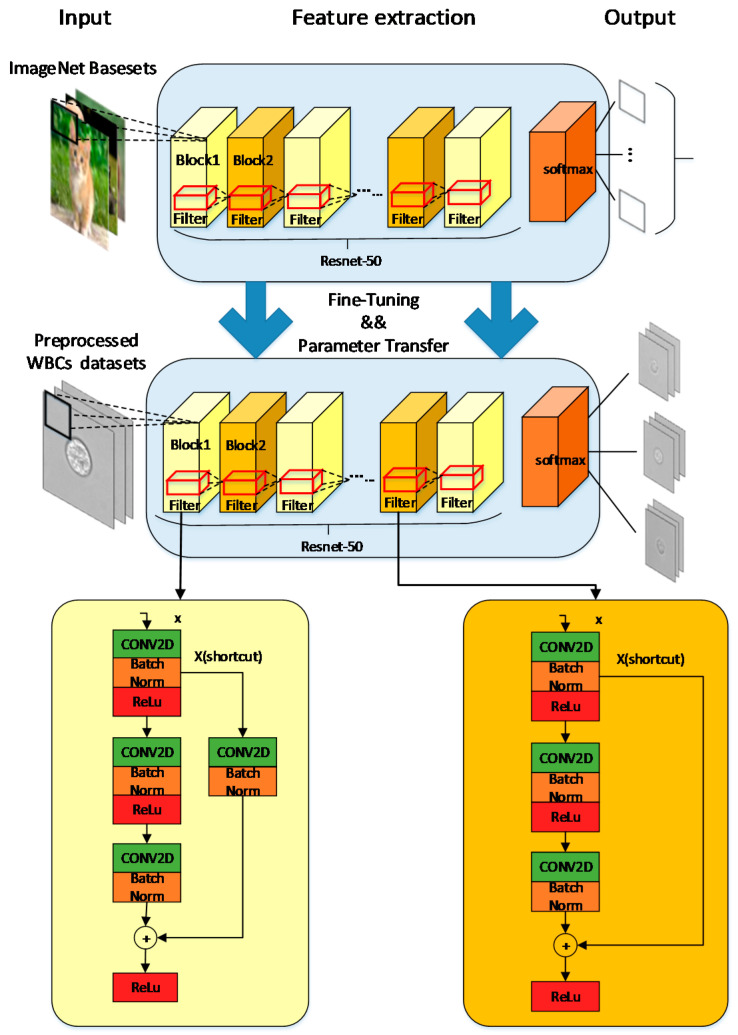
Diagram of Resnet-50 neural network with Identity Block shown on the bottom left and Conv Block shown on the bottom right.

In this paper, we used WBC image datasets, including 10,400 Monocytes, 10,392 Lymphocytes, and 10,266 Granulocytes. Among them, 80% of the WBC image datasets were used as the training set, while 20% were used as the testing set. The whole processing flow is shown in Figure 2. The Resnet-50 network was used in our task for transfer training [21], whose structure included two basic blocks, as shown in Figure 4. Resnet-50 network adds a residual unit through a short connection system.

The advantages of joining the residual unit network are as follows: First, the training process connects the network characteristics of different layers. Using the globalization of the Resnet-50 network can better classify the label-free WBC images because they have more details than labeled WBC images, which typically only show the features of the nucleus. Second, the short connection mechanism can suppress phenomena, such as gradient disappearance, gradient explosion, overfitting, and network degradation. Resnet-50’s short connection makes it more “flexible” during training. The work can choose between “more convolution and nonlinear transformation” (connection) or “more inclined to do nothing” (skip), or a combination of both. “Flexible” Resnet-50 network structure can better adapt to the polymorphic label-free leukocytes. Previous work using this short connection also achieved a good effect on the feature extraction of WBC [14]. Third, the residual block in the Resnet-50 network can make the network easier to train and get better weight values. It relatively eliminates the parameters of each layer in the network so that the training speed becomes faster.

There are also some other typical deep learning models, such as Inception V3 or Resent-101. Compared with the Inception V3 model without short connection structures, the short connection structure of Resnet-50 is more suitable for feature extraction of label-free WBCs. Resnet-101 and Resnet-50 only differ in the number of layers. Considering the number of datasets and only three classifications we selected, a complex classification model would cause overfitting and increase the training time. For label-free WBC classification, too many layers in the model will cause overfitting. Therefore, the Resnet-50 network was selected as the WBC three-type classification in our study.

The Resnet-50 network and transfer learning were implemented through a pre-trained model under the TensorFlow framework. Training based on pre-trained models will reduce training time and prevent overfitting. The weight value in the selected pre-training model was close to the local optimal value. Therefore, the pre-training weight value can be kept in the high gradient range in future training for effective adjustment. The last layer of the pre-training model was removed, and the remaining parameters were reloaded, then we continued training with the output of three type classifications. The original Resnet-50 is suitable for three-channel images. But the WBC images were single-channel grayscale images, so the datasets were superimposed to convert single-channel grayscale images to three-channel grayscale images. In the model, we added dropout and learning rate, decreasing optimization methods. The dropout method can increase the sparsity of the model and reduce overfitting. The learning rate decreasing method will gradually reduce the learning rate during training, which can reduce the training time and find the best learning rate that maximizes the gradient descent effect. If the learning rate remains the same, it may cause the loss value to stop decreasing without reaching the minimum. The gradient descent is a deep learning algorithm to continuously and iteratively update the model. The Root Mean Square Prop (RMSProp) algorithm was used in the model as an optimizer for gradient descent, and it solved the problem of excessive swing in the optimization loss and further accelerated the convergence speed [22].

We trained 2000 steps, set batch size to 256, dropout value to 0.5, and basic learning rate to 0.001. For every 100 steps, the learning rate dropped to 0.99 times the base learning rate. The activation function was Rectified Linear Unit (ReLU), and the objective function to be minimized was (2),

(2) $$\omega^{*}=\arg\begin{array}{c} min\\ \omega \end{array}\underset{i}{\sum }L\left(y_{i},f\left(x_{i}\right)\right)$$

The Loss value formula is shown in (3), where $\;y_{i}$ represents the label input by the model (real sample), $x_{i}$ represents the input of the model, $f\left(x_{i}\right)$ represents the predicted value of the model output, ω represents the weight value of the model, $\;\omega^{*}$ represents the new weight value after the model has been updated.

(3) $$L=\;\frac{1}{N}\underset{i}{\sum }L_{i}=\;\frac{1}{N}\underset{i}{\sum }\;-\;\overset{M}{\underset{c=1}{\sum }}y_{\mathrm{ic}}\log\left(p_{\mathrm{ic}}\right)$$

In (3), M represents the number of categories, N represents the total sample size, ${\;y}_{\mathrm{ic}}$ represents the indicator variable. If the current category is the same as the sample i category, $y_{\mathrm{ic}}$ = 1; otherwise $y_{\mathrm{ic}}$ = 0. $p_{\mathrm{ic}}$ represents the predicted probability that sample i belongs to class sample c.

### 2.4. Activated and Inactivated Neutrophil Classification

The neutrophils can be classified into activated and inactivated states based on the cell morphologies. Inactivated neutrophils are spherical. Once there is biochemical stimulation or mechanical stimulation, there will be obvious morphological changes, changing from round to worm (some contain synapses) and become activated. Consider the characteristics of the morphological changes of neutrophils after activation, we used the aspect ratio and roundness of the cell to distinguish between activated and inactivated neutrophils. The aspect ratio is the ratio between length and width, as shown in (4),

(4) $$\;\mathrm{Aspect}\;\mathrm{Ratio}=\;\frac{x}{y}$$

where *x* represents length and *y* represents width (the longer one has a diameter as long in the default image, and the shorter one has a wide diameter). Roundness is based on the ratio of the measured cell area to the circumference to determine the similarity between the cell and the circle, as shown in (5),

(5) $$\mathrm{Roundness}=\;\frac{4\;*\;\pi \;*\;area}{perimeter^{2}}$$

where the area represents the area of the image, and the perimeter represents the perimeter of the image.

For inactivated neutrophils, aspect ratio and roundness are both close to 1 [5]. But activated neutrophils will be different from inactivated neutrophils in aspect ratio and roundness value. In this paper, when calculating the aspect ratio and roundness value of the cell, convolution filtering was performed on the WBC images. The convolution kernels used in convolution filtering were *K*1 and *K*2:

$$K1=\left[\begin{array}{ccc} -1 & -1 & -1\\ 0 & 0 & 0\\ 1 & 1 & 1 \end{array}\right],\;K2=\left[\begin{array}{ccc} -1 & 0 & 1\\ -1 & 0 & 1\\ -1 & 0 & 1 \end{array}\right]$$

After filtering, the same segmentation method was used as above to obtain the WBC contour coordinates, determine the length and width of WBCs, and the size of the perimeter area, and finally calculate the aspect ratio and roundness value. For the cells with an aspect ratio greater than 1.2, it was identified as an activation state. If it was less than 1.2, the roundness was further judged. When the roundness value was greater than 0.76, the WBC was identified as in the activated state. Otherwise, it was identified as in the inactivated state. Some examples of the morphological analysis are shown in Table 2. As the granulocyte classified by our model contained three types: basophil, neutrophil, and eosinophil, and basophils and eosinophils accounted for only about 5% of granulocytes, we treated the granulocytes as neutrophils when doing the morphological analysis for activated and inactivated neutrophils. The results were then corrected by the proportion of basophil, eosinophil, and neutrophilic granulocytes. The previous work also mentions the method of using proportional numbers to correct the results [23]. Through color adjustment, threshold segmentation, and spot detection methods, the RBC, WBC, and platelets in the blood were separately identified and counted, then the final count of each WBC type was proportionally obtained.

## 3. Results

We segmented the WBC images, extracted the features of the WBCs from the training set through the Resnet-50 network, and classified them into three categories. The training set was composed of 8621 Granulocytes, 8384 Lymphocytes, and 8391 Monocytes. After every 100 steps of training, the testing sets were used to test the model, and the 200 steps of training were used to save and evaluate the model. Then, the classified granulocyte images were further identified as activated state or inactivated state through morphological examination.

This training was carried out under a 64-bit Linux system, using Nvidia Kepler K80 GPU accelerator card, an eight-channel high-performance SAS RAID card (4 GB cache), and 16 G ECC Registered DDR4 2133 memory. The code was written using Python 2.7, TensorFlow 1.13.1 version.

We used a confusion matrix to obtain *Precision*, *Recall*, and *F*1_*Score* for evaluation. The confusion matrix was used to summarize the result of a classifier. For classification, the confusion matrix was a k*k table, which was used to record the relationship between the predicted result and the actual label. The *Recall* formula is shown in (6), which represents the probability of being predicted as a positive sample in a sample that was positive. The formula for the *Precision* is shown in (7), which represents the probability of a positive sample among all the samples predicted to be positive. The higher *Recall* and *Precision* are, the better the classification results we get, but they are also a pair of contradictions. *F*1_*Score*, as shown in (8), is a trade-off between them. Both *Precision* and *Recall* estimate positive samples, and the overall estimate still uses *Accuracy* as the standard. The formula of *Accuracy* is as shown in (9). Among them, True Positive (*TP*) means positive samples predicted to be positive, True Negative (*TN*) means negative samples predicted to be negative, False Positive (*FP*) means negative samples predicted to be positive, and False Negative (*FN*) represents a positive sample predicted to be negative. We also used 95% confidence intervals (CI) to evaluate the reliability of *Accuracy*. The formula of CI is as shown in (10), where n represents the number of *Accuracy* values selected, $\;\hat{\mu }$ represents the average of selected *Accuracy* values, σ represents the standard deviation of selected *Accuracy* values, and M represents the number of 95% confidence intervals. We also applied the Receiver Operating Characteristic (ROC) curve to evaluate the model, where the vertical axis of the ROC curve is True Positive Rate (*TPR*) as in (11), and the horizontal axis is False Positive Rate (*FPR*) as in (12). The ROC curve can accurately reflect the relationship between *TP* and *FP*, which is a comprehensive representation of the detection accuracy. The ROC curve expresses the correctness of the selected eigenvalues. The closer the deviation of the curve to the (0,1) point, the more successful the feature value selection is. The Area Under the ROC Curve (AUC) value refers to the area of the ROC curve. The larger the AUC value is, the more successful model feature value selection is.

(6) $$Recall=\;\frac{TP}{TP+FN}$$

(7) $$Precision=\;\frac{TP}{TP+FP}$$

(8) $$F1\_Score=\;\frac{2*Precision*Recall}{Precision+Recall}$$

(9) $$Accuary=\;\frac{TP+TN}{TP+TN+FP+FN}$$

(10) $$P\left(\hat{\mu }-1.96\frac{\sigma }{\sqrt{n}}\leq M\leq \hat{\mu }+1.96\frac{\sigma }{\sqrt{n}}\right)\approx 0.95$$

(11) $$TPR=\;\frac{TP}{TP+FN}$$

(12) $$FPR=\;\frac{FP}{TP+TN}$$

**Figure 5:**
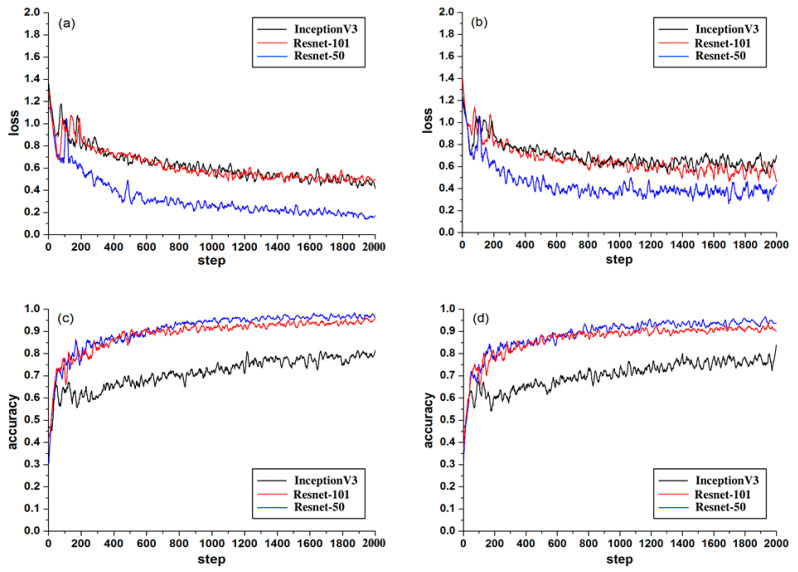
Comparison results of Loss and Accuracy during training and testing for Inception V3, Resnet-101, and Resnet-50: (**a**) is the training loss, (**b**) is the testing loss, (**c**) is the training accuracy, and (**d**) is the testing accuracy.

After 2000 steps of training, it was finally concluded that the accuracy of the Resnet-50 training set was about 95%, and the accuracy of the Resnet-50 testing set could reach more than 90%. The 95% confidence interval of the testing set was [0.943 − 0.004, 0.943 + 0.004]. Testing accuracy was close to the training accuracy, indicating that there was no overfitting phenomenon in Resnet-50. The value of Accuracy was relatively stable in both testing and training sets, so it proved the effectiveness of the Resnet-50 model. Besides, the Recall and Precision of the Resnet-50 testing set were about 90% and 96%, respectively, F1_Score of the testing set was about 93%, which proved that the relationship between Recall and Precision was relatively balanced. The curves of Loss and Accuracy are shown in Figure 5. After 1000 steps, the loss value of the Resnet-50 testing set fluctuated greatly around 0.3. This could be caused by the fact that the label-free WBC images have more feature values and less regular than the fluorescently labeled WBCs.

We used two other transfer learning models for comparative analysis, including InceptionV3 network and Resnet-101 network with pre-trained models (the pre-trained models are all from ImageNet). As a classic network model, InceptionV3 has been applied to classification tasks in many works. The only difference between Resnet-101 and Resnet-50 is the number of their layers.

**Figure 6:**
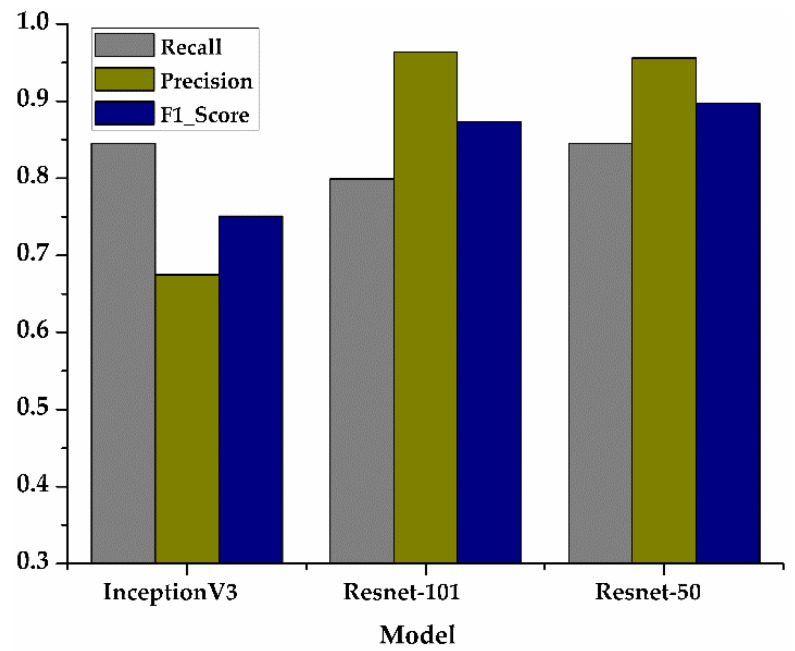
Comparison results of Recall, Precision, F1_score for three models.

**Figure 7:**
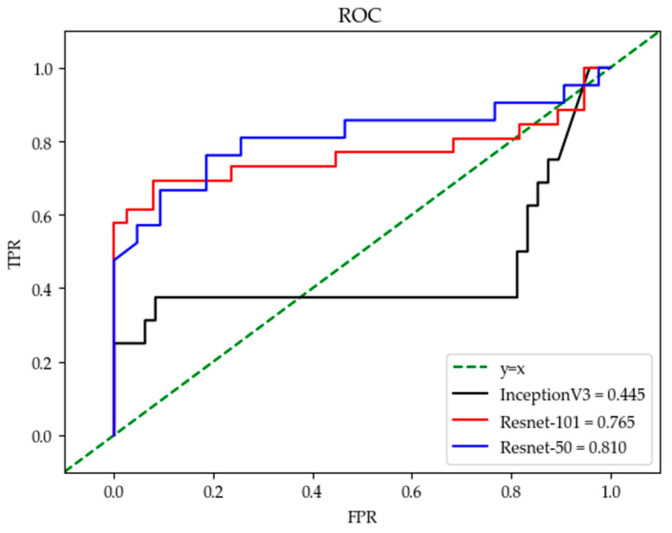
Comparison results of ROC curves for the three models.

**Figure 8:**
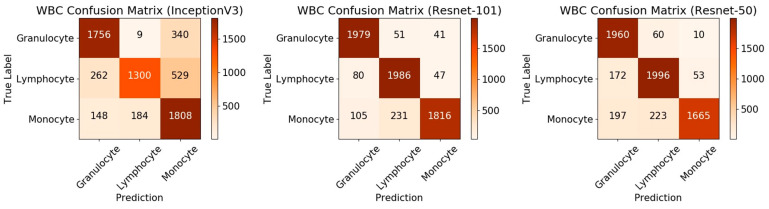
Comparison results of the confusion matrix for the three models.

From the comparison of the loss values of the training set and the testing set in Figure 5a,b, it can be seen that the loss value of the Resnet-50 model was the lowest. In the accuracy comparison of the training set and testing set of Figure 5c,d, although the accuracy of Resnet-50 and Resnet-101 was almost the same, it can be seen from the comparison value of the three models in Figure 6 that the F1_score of Resnet-50 was the highest, which proved that the overall performance of Resnet-50 was better than the other two models. In addition, the 95% confidence interval of the Resnet-50, Resnet-101, and InceptionV3 are shown in Table 3. The fluctuation of Resnet-50 ( ±0.004 ) was less than the Resnet-101 ( ±0.005 ) and the InceptionV3 ( ±0.008 ), which proved that the accuracy of Resnet-50 was more stable than the other two models. It can also be seen in the comparison of the ROC curve as in Figure 7 that the curve of Resnet-50 was the closest to the (0,1) point. The straight-line y = x in Figure 7 represents the result of a classifier using a random guessing strategy. It can be seen that the Resnet model was on the ROC curve above the y = x straight line, which proves that the Resnet model was better than the randomly guessed classifier. The reason for the poor performance of the InceptionV3 model could be that there is no residual network structure in the model and the feature value extraction is not accurate enough. Resnet-101 is deeper than Resnet-50, but the loss value was not lower than Resnet-50, and the value of F1_Score and AUC were not better than Resnet-50, indicating that increasing more layers may not be effective. From the data of the confusion matrix in Figure 8, it can be seen that the classification accuracy of granulocyte (95.6%) and lymphocyte (94%) were high, while the monocyte (84.3%) was low. It indicated that the characteristics of monocyte cells after training were not very obvious and were easily misidentified as lymphocytes. The above results prove that it is feasible to use the Resnet-50 network with a pre-training model to realize a three-type classification for WBCs.

Based on the automatic classification and counting of the activated and inactivated WBCs, a total of 1771 granulocyte cells were tested, the number of activated cells obtained was 365, and the number of inactivated cells was 1328. According to the ratio of basophil and eosinophil, they account for 1% and 5% of the entire granulocytes, respectively. Therefore, the number of activated neutrophils was about 346, and the number of inactivated neutrophils was about 1261.

## 4. Discussions

Similar work [24] has used the similar Resnet-18 model to classify label-free WBCs (neutrophils and monocytes) with the highest accuracy of 64.9%, but the accuracy of our method reached about 90%, as shown in Table 3. The article pointed out that the reason for the unsatisfactory model was the imbalance of the dataset and low resolution of the obtained images. The impact of dataset imbalance on Accuracy is also reflected in the Resnet-50 model: we found that the Recall of monocyte was not very high, so it was sometimes recognized as a lymphocyte, but other types of WBCs did not have such problems. This could be due to the imbalance of the initial dataset since the monocyte in the human blood at the time of sampling is less. Although we have expanded the dataset, the real features of the monocyte were still less than the other two classes, so the accuracy of the monocyte was relatively low.

There are probably three reasons for our higher accuracy results. First, the WBC image resolution used was higher (obtained by a 100× objective microscope), compared with the cell resolution taken by flow cytometry in [23], the resolution of our dataset was much higher. Second, the Resnet-50 model we used had more layers than the Resnet-18 model, so the extracted feature values were more accurate. Third, the pre-training model we added was helpful to improve the accuracy. Compared with the deep learning model that uses stained WBC images as a dataset, the results of our model are also impressive. In previous work [25], the four-layer model built by itself was used to classify the stained WBCs, in which the obtained Accuracy and Recall were similar to our experimental results. Previous work [26] also used deep learning methods to extract features of stained WBCs, the author used Lenet-5, Alexnet model for comparison, and finally used the self-built WBCsNet structure to get the highest accuracy rate of 93%. During the experiment, we also tried to use Lenet-5 and Alexnet’s migration learning models for three classifications. Because the feature values of label-free cells are more difficult to extract, the effect was not good for models with fewer layers. Our classification results based on unstained WBC were similar to those based on stained WBC, indicating the feasibility of our label-free WBC classification method.

However, our method of extracting feature values using the Resnet-50 model can still be further improved. In [27], it proposed the use of the IDEAS tool to extract the characteristic values of five types of WBCs, and the accuracy reached was about 97%. Similarly, in [17], the proposed manual extraction of feature values method to classify WBCs had an accuracy of 99%. Compared with machine learning methods, Resnet-50 is not very accurate in extracting feature values, resulting in the classification accuracy being not as high as using machine learning or even manual classification. Therefore, the automatic extraction of WBC features using deep learning requires further improvement. Although the accuracy of the Resnet-50 model is not prominent, it can be used as a method of initial medical diagnosis. In some remote and resource-limited places where cell sorting equipment or manual sorting conditions are lacking, this method provides a convenient solution.

In terms of the method used in this paper to distinguish activated and inactivated neutrophils, it was based on obvious changes in morphological characteristics. The ideal result can be obtained by simple convolution filtering and calculation of cell characteristics on the cell image with less time consumption. Its advantages are high speed and low requirements for computer performance. The fast testing speed can be reflected in the testing of 1771 cell images in only 60 s. If the deep learning method is used for testing, the training process and the test process will take a long time, and the computer configuration requirements are relatively high. The lower error rate is also a major advantage of this method. If deep learning or machine learning is used for two-class training, it will increase the difficulty and time consumption. So this is a more convenient and concise method. The disadvantage is that the total number of cells tested was 1771, but the total number of activated cells and inactivated cells was only 1693, which proves that this system was insufficient to identify the contours of cells because some cells were not detected, so that the predicted number was smaller than the total number before the test. In this respect, further improvement is still needed.

## 5. Conclusions

This paper proposed a label-free three-type WBC classification method using the transfer learning technique based on the Resnet-50 neural network. The features of label-free WBCs could be extracted and divided into three categories, such as granulocyte, lymphocytes, and monocytes. The activated and inactivated neutrophils, which are the dominant granulocytes, could be detected for immune state monitoring. The Resnet-50 model-based method was faster and more convenient than other machine learning or manual detection methods. The accuracy of this article can reach about 90%, which verifies the feasibility of using label-free WBC images for classification. Although the accuracy is not as high as that of manually extracting feature values using machine learning, our method is more convenient and time-saving. The research in this article is helpful for the development of label-free cell classification methods. Using label-free cell images for classification is becoming a future trend. In future work, we will improve the network so that it will be more suitable for the extraction of label-free WBC feature values to increase the accuracy of the results.

## Figures and Tables

**Table 1:** Comparison of white blood cells (WBC) datasets before and after enhancement.

WBC Type	Monocyte	Lymphocyte	Granulocyte
Segmentation	211	444	1540
Enhancement	10,400	10,392	10,266

**Table 2:** The identification process of activated or inactivated WBCs.

Original Image	Convolution Filtering	Mask Image	Contour Image	Aspect Ratio	Roundness	Status
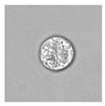	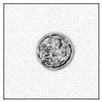	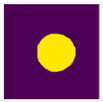	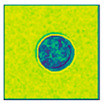	1	0.89	Inactivated
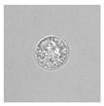	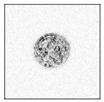	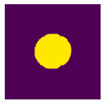	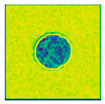	1.04	0.86	Inactivated
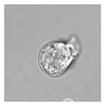	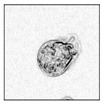	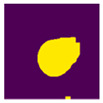	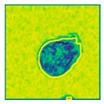	1.22	0.64	Activated
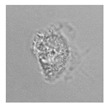	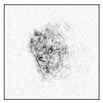	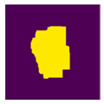	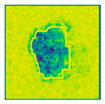	1.34	0.67	Activated

**Table 3:** Comparison of Confidence Intervals (CI) and Average Accuracy for different models.

Model	95% Confidence Intervals	Average Accuracy
Resnet-18	--	0.649
InceptionV3	[0.767 − 0.008, 0.767 + 0.008]	0.767
Resnet-101	[0.9126 − 0.005, 0.9126 + 0.005]	0.9126
Resnet-50	[0.943 − 0.004, 0.943 + 0.004]	0.943

## Data Availability

Data available on request due to privacy restrictions.
