# Correction: Huang et al. Deep-Learning Based Label-Free Classification of Activated and Inactivated Neutrophils for Rapid Immune State Monitoring. *Sensors* 2021, *21*, 512

**DOI:** 10.3390/s21248360

**Published:** 2021-12-15

**Authors:** Xiwei Huang, Hyungkook Jeon, Jixuan Liu, Jiangfan Yao, Maoyu Wei, Wentao Han, Jin Chen, Lingling Sun, Jongyoon Han

**Affiliations:** 1 Key Laboratory of RF Circuits and Systems, Ministry of Education, Hangzhou Dianzi University, Hangzhou 310018, China; hduljx@hdu.edu.cn (J.L.); yaojiangfan@hdu.edu.cn (J.Y.); lucienwell@hdu.edu.cn (M.W.); wentaohan@hdu.edu.cn (W.H.); chenjin272@hdu.edu.cn (J.C.); sunll@hdu.edu.cn (L.S.); 2 Research Laboratory of Electronics, Massachusetts Institute of Technology, Cambridge, MA 02139, USA; hkjeon@mit.edu (H.J.); jyhan@mit.edu (J.H.); 3 Department of Electrical Engineering and Computer Science, Massachusetts Institute of Technology, Cambridge, MA 02139, USA; 4 Department of Biological Engineering, Massachusetts Institute of Technology, Cambridge, MA 02139, USA

The authors wish to make the following correction to their paper [1]:

Hyungkook Jeon and Jongyoon Han have been added as co-authors due to their contribution in processing blood samples using microfluidic blood sorting devices.

The revised authorship, affiliation and funding are now as follows:

Xiwei Huang ^1,^*^,†^, Hyungkook Jeon ^2,†^, Jixuan Liu ^1^, Jiangfan Yao ^1^, Maoyu Wei ^1^, Wentao Han ^1^, Jin Chen ^1^, Lingling Sun ^1^ and Jongyoon Han ^2,3,4^

^1^  Key Laboratory of RF Circuits and Systems, Ministry of Education, Hangzhou Dianzi University, Hangzhou 310018, China; hduljx@hdu.edu.cn (J.L.); yaojiangfan@hdu.edu.cn (J.Y.); lucienwell@hdu.edu.cn (M.W.); wentaohan@hdu.edu.cn (W.H.); chenjin272@hdu.edu.cn (J.C.); sunll@hdu.edu.cn (L.S.)
^2^  Research Laboratory of Electronics, Massachusetts Institute of Technology, Cambridge, MA 02139, USA; hkjeon@mit.edu (H.J.); jyhan@mit.edu (J.H.)
^3^  Department of Electrical Engineering and Computer Science, Massachusetts Institute of Technology, Cambridge, MA 02139, USA
^4^  Department of Biological Engineering, Massachusetts Institute of Technology, Cambridge, MA 02139, USA
*  Correspondence: huangxiwei@hdu.edu.cn
^†^  These authors contributed equally to this work.
